# Supplementary material for: Folic Acid Improves Parkin-Null Drosophila Phenotypes and Transiently Reduces Vulnerable Dopaminergic Neuron Mitochondrial Hydrogen Peroxide Levels and Glutathione Redox Equilibrium
Source: Antioxidants (Basel). 2022 Oct 20;11(10):2068. doi: 10.3390/antiox11102068 (PMC9598960; doi:10.3390/antiox11102068)
Supplement: Supplementary file 1 [file antioxidants-11-02068-s001.zip › antioxidants-1934174-supplementary.pdf]

antioxidants

Supplementary figure captions

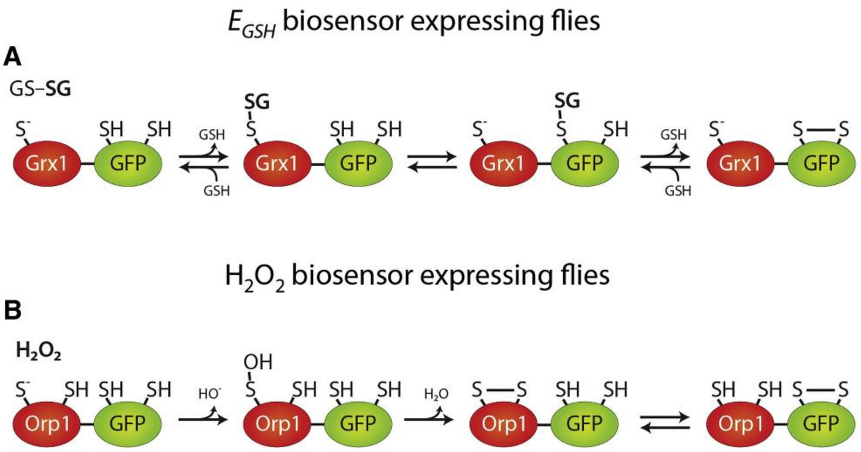

Figure S1. Cartoon of mechanisms by which (A) roGFP2-Grx1 and (B) roGFP2-Orp1 detect glutathione redox equilibrium and hydrogen peroxide. Used with permission from Morgan et al., 2011 (DOI: 10.1016/j.freeradbiomed.2011.08.035; Elsevier (License number 5401470003088).

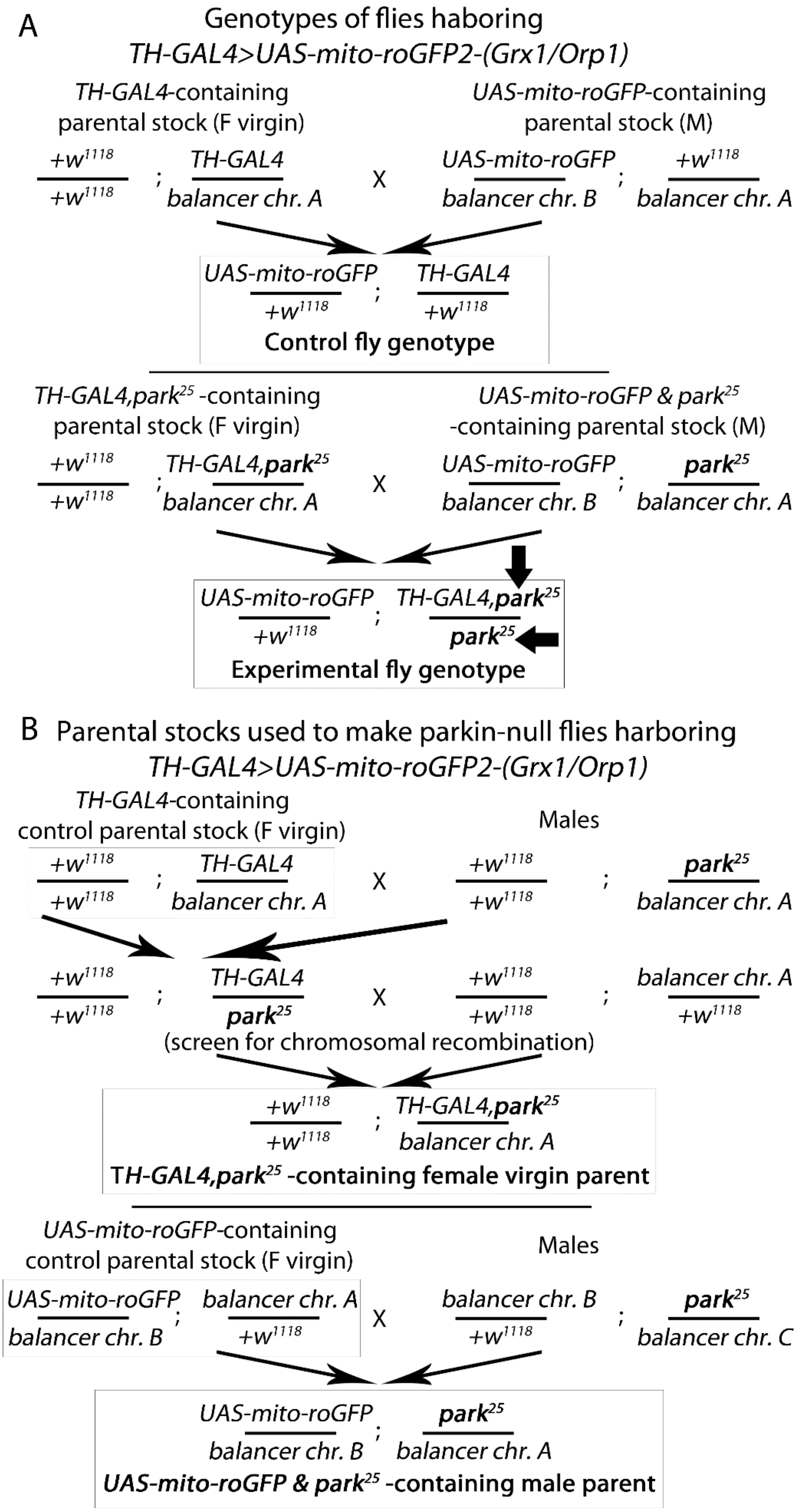

Figure S2. Strategy for generating parkin-null flies that drive expression of mito-roGFP2-Grx1 and mito-roGFP2-Orp1 into *Drosophila* dopaminergic neurons. (A) transgene-containing chromosomes and their sister chromosomes for parental stocks are shown for control and parkin-null flies. Sister

chromosomes are separated by a horizontal line, and chromosome pairs are separated by a semicolon. An "X" indicates mating or "crossing" of female and male flies. *Park*^{25} is the excision mutant generated by Leo Pallanck at the University of Washington, Seattle (Greene et al., 2003). Black boxes indicate experimental genotypes. Block arrows and bold font indicate the difference in genotypes used in this study. (B) Crossing scheme for generation of parkin-null parental stocks used in A by introducing the parkin-null *park*^{25} allele into control parental genotypes. *TH-GAL4*-containing and UAS-mito-roGFP2-(Grx1 or Orp1)-containing control parental stocks are indicated in blue and purple boxes, respectively. *TH-GAL4*-containing and UAS-mito-roGFP2-(Grx1 or Orp1)-containing parkin-null parental stocks are indicated in green and yellow boxes, respectively. "+w^{1118}" indicates a chromosome that is crossed in from a standard control fly genotype. Balancer chromosomes ("balancer chr.") are engineered chromosomes that are homozygous lethal and that cause flies to have easily identifiable wing or bristle features. *TH-GAL4* and *UAS* constructs confer identifiable eye color to aid in progeny selection.

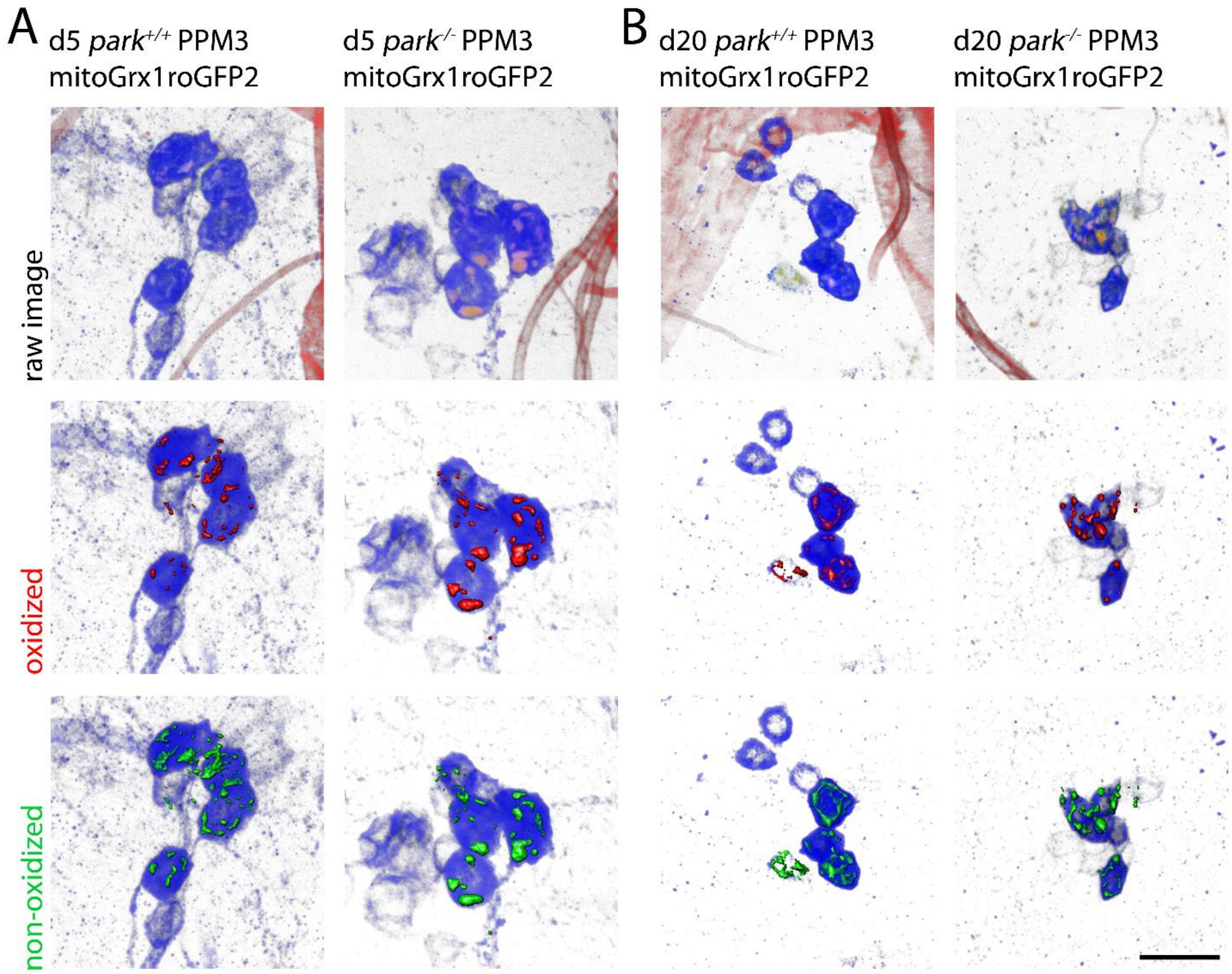

Figure S3. Representative images mito-roGFP2-Grx1 expression in PPM3. Brains were dissected from control (*park*$^{+/+}$) or parkin-null (*park*$^{-/-}$) flies expressing mito-roGFP2-Grx1 on days 5 and 20 post-eclosion, and ratios of total volumes of oxidized to non-oxidized fluorophore emissions were calculated for one PPM3 region per brain. Representative panels of PPM3 images with mito-roGFP2-Grx1 on days (A) 5 and (B) 20. Raw images are shown in the top row, where blue indicates tyrosine hydroxylase antibody labeling, and red and green indicate oxidized and non-oxidized roGFP2, respectively. Selected volume "isosurfaces" of oxidized (red, middle row) and non-oxidized (green, bottom row) mito-roGFP2-Grx1 above threshold are also shown. Scale bar, 10 μm.

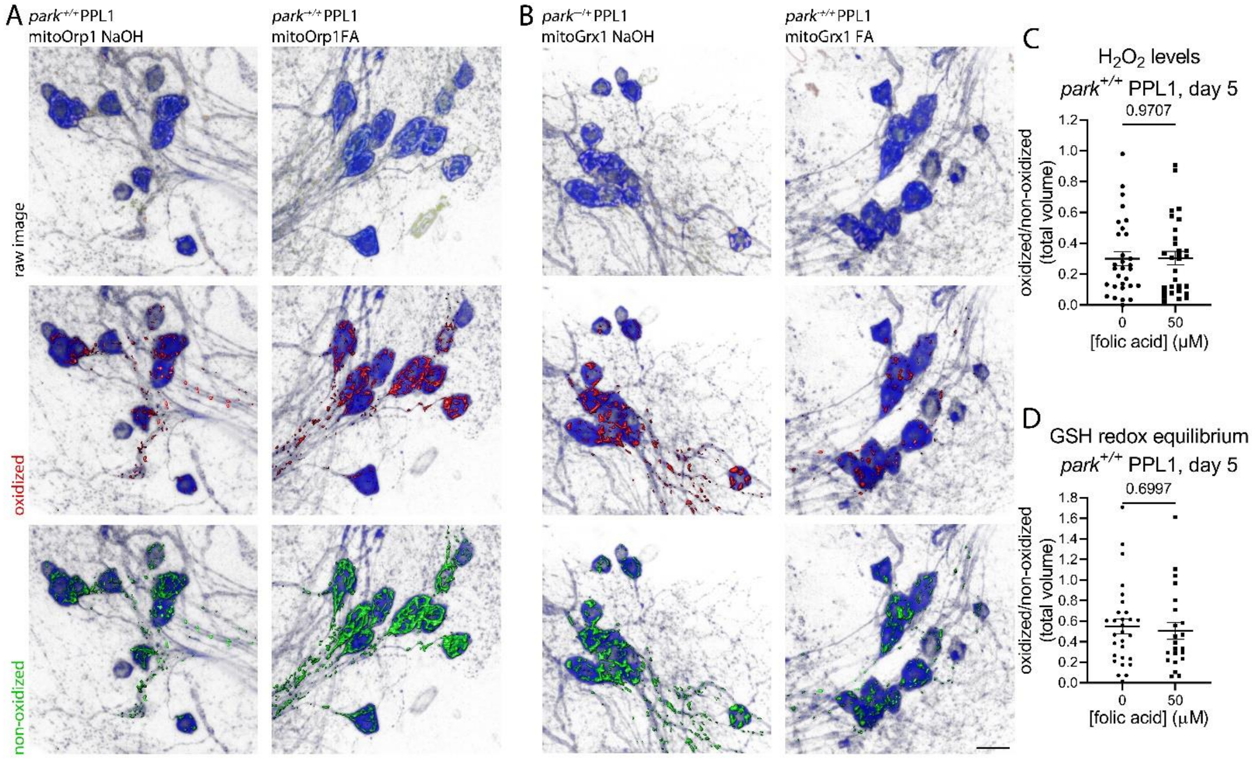

Figure S4. Folic acid administration does not affect PPL1 mitochondrial hydrogen peroxide levels or glutathione redox equilibrium in day 5 control flies. Brains were dissected from folic acid-treated *park*$^{+/+}$ flies expressing (A) mito-roGFP2-Orp1 or (B) mito-roGFP2-Grx1 on day 5 post-eclosion, and ratios of total volumes of oxidized to non-oxidized fluorophore emissions were calculated for one PPL1 region per brain. Representative raw images are shown in the top row, where blue indicates tyrosine hydroxylase antibody labeling, and red and green indicate oxidized and non-oxidized roGFP2, respectively. Selected volume "isosurfaces" of oxidized (red, middle row) and non-oxidized (green, bottom row) (A) mito-roGFP2-Orp1 and (B) mito-roGFP2-Grx1 above threshold are also shown. Scale bar, 10 µm. (C, D) Each data point represents the ratio from one PPL1 region. Error bars represent standard error of the mean, and P values are reported for each comparison ($n \geq 22$).

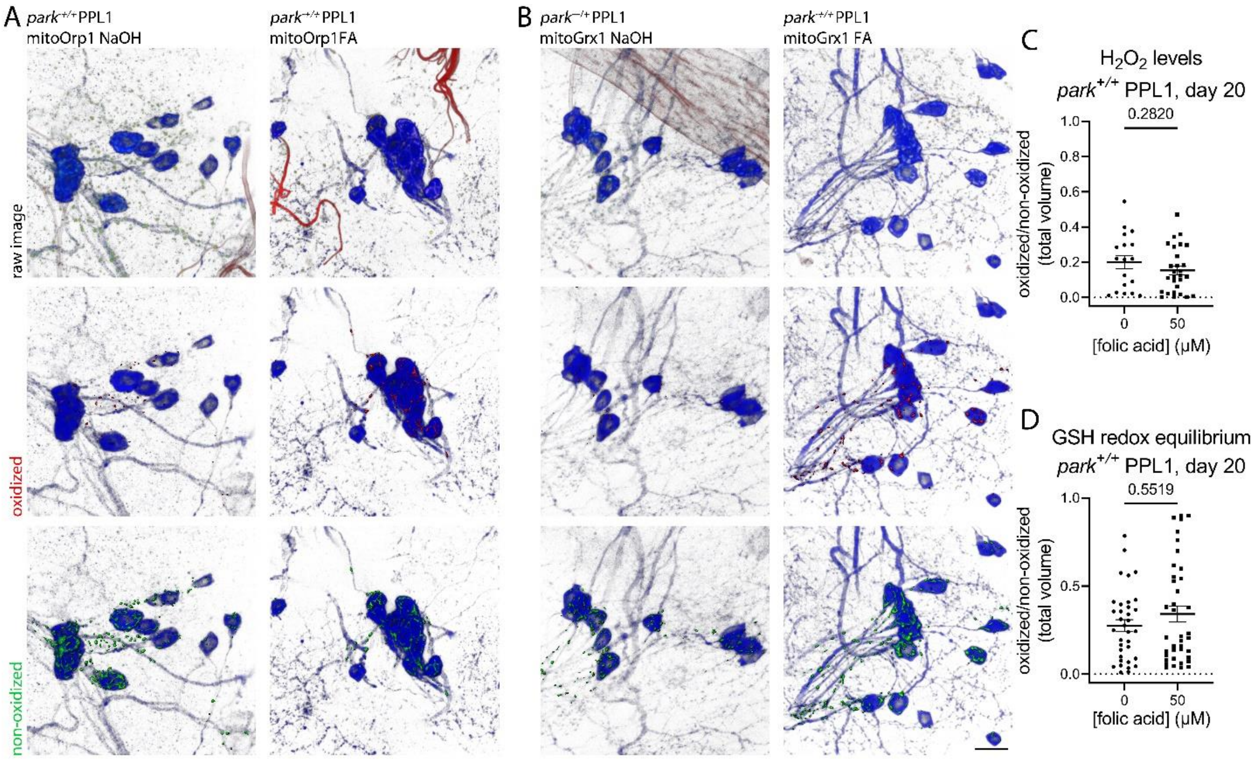

Figure S5. Folic acid administration does not affect PPL1 mitochondrial hydrogen peroxide levels or glutathione redox equilibrium in day 20 control flies. Brains were dissected from folic acid-treated $park^{+/+}$ flies expressing (A) mito-roGFP2-Orp1 or (B) mito-roGFP2-Grx1 on day 20 post-eclosion, and ratios of total volumes of oxidized to non-oxidized fluorophore emissions were calculated for one PPL1 region per brain. Representative raw images are shown in the top row, where blue indicates tyrosine hydroxylase antibody labeling, and red and green indicate oxidized and non-oxidized roGFP2, respectively. Selected volume "isosurfaces" of oxidized (red, middle row) and non-oxidized (green, bottom row) (A) mito-roGFP2-Orp1 and (B) mito-roGFP2-Grx1 above threshold are also shown. Scale bar, 10 μm. (C, D) Each data point represents the ratio from one PPL1 region. Error bars represent standard error of the mean, and P values are reported for each comparison (n ≥ 18).

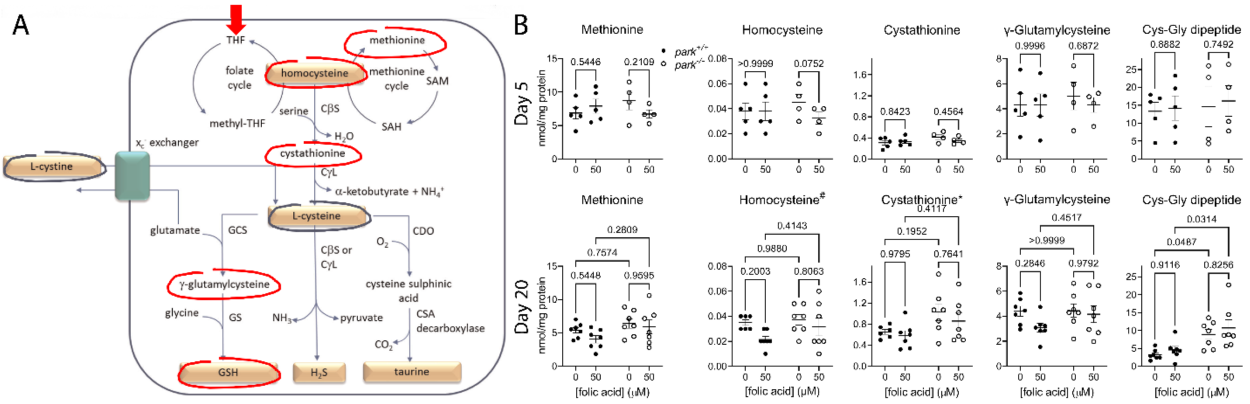

Figure S6. Liquid chromatography tandem mass spectrometry measurements of glutathione synthesis pathway molecules in day 5 and 20 control and parkin-null heads. (A) Diagram depicting the role of folic acid (tetra hydro folate [THF], red arrow) in glutathione (GSH), H_2S, and taurine synthesis. Measured markers of GSH synthesis are circled in red. Cystine and cysteine, measured in figure 7, are circled in grey. (B) Heads of folic acid-treated $park^{+/+}$ and $park^{-/-}$ flies were collected and frozen on days 5 (top row) and 20 (bottom row) post eclosion. LC-MS/MS analysis was performed to measure levels of methionine, homocysteine, cystathionine, γ-glutamylcysteine, and cysteinyl-glycine dipeptide, a marker of glutathione catabolism (not shown in A). Folic acid administration decreases homocysteine levels on day 20, while cystathionine and cysteinyl-glycine dipeptide were

increased in parkin-null flies. Each data point represents one tube of lysate or the average of up to three lysate tubes collected on the same day, each tube containing seven to thirty-four heads. Effects of genotype and folic acid were determined using two-way repeated measures ANOVA followed by Šídák's multiple comparison test (day 5) or two-way ANOVA followed by Tukey's multiple comparisons test (day 20). For graph titles, "#" indicates a significant effect of folic acid administration, and "*" indicates a significant effect of genotype ($P < 0.05$ for both). P values for post hoc comparisons are shown ($n = 5$ for day 5 and 7 for day 20).
